# Why do patients interrupt and return to antiretroviral therapy? Retention in HIV care from the patient’s perspective in Johannesburg, South Africa

**DOI:** 10.1371/journal.pone.0256540

**Published:** 2021-09-02

**Authors:** Melanie A. Bisnauth, Natasha Davies, Sibongile Monareng, Fezile Buthelezi, Helen Struthers, James McIntyre, Kate Rees

**Affiliations:** 1 Anova Health Institute, Johannesburg, South Africa; 2 School of Public Health and Family Medicine, University of Cape Town, Cape Town, South Africa; 3 Division of Infectious Diseases & HIV Medicine, Department of Medicine, University of Cape Town, Cape Town, South Africa; 4 Department of Community Health, School of Public Health, University of the Witwatersrand, Johannesburg, South Africa; Emory University School of Medicine, UNITED STATES

## Abstract

**Background:**

Retention in care is required for optimal clinical outcomes in people living with HIV (PLHIV). Although most PLHIV in South Africa know their HIV status, only 70% are on antiretroviral therapy (ART). Improved retention in care is needed to get closer to sustained ART for all. In January 2019, Anova Health Institute conducted a campaign to encourage patients who had interrupted ART to return to care.

**Methods:**

Data collection was conducted in one region of Johannesburg. This mixed methods study consisted of two components: 1) healthcare providers entered data into a structured tool for all patients re-initiating ART at nine clinics over a nine-month period, 2) Semi-structured interviews were conducted with a sub-set of patients. Responses to the tool were analysed descriptively, we report frequencies, and percentages. A thematic approach was used to analyse participant experiences in-depth.

**Results:**

562 people re-initiated ART, 66% were women, 75% were 25–49 years old. The three most common reasons for disengagement from care were mobility (30%), ART related factors (15%), and time limitations due to work (10%). Reasons for returning included it becoming easier to attend the clinic (34%) and worry about not being on ART (19%). Mobile interview participants often forgot their medical files and expressed that managing their ART was difficult because they often needed a transfer letter to gain access to ART at another facility. On the other hand, clinics that had flexible and extended hours facilitated retention in care.

**Conclusion:**

In both the quantitative data, and the qualitative analysis, changing life circumstances was the most prominent reason for disengagement from care. Health services were not perceived to be responsive to life changes or mobility, leading to disengagement. More client-centred and responsive health services should improve retention on ART.

## Background

Retention in care (RIC) is required for optimal clinical outcomes in people living with HIV. To reach the UNAIDS second and third 90 targets, keeping people who are on antiretroviral therapy (ART) engaged in care is one of the most important tasks health systems must achieve [1–9]. In the context of universal antiretroviral therapy (ART), once a patient has accessed medical care and is receiving ART at the end of a follow-up period, they are considered to be retained in care [1]. In healthcare settings, visit frequency has often been used as a measure of RIC [1, 3, 10, 11]. In the HIV programme in South Africa, and for the purpose of this article, loss to follow-up is defined as a patient who has missed their appointment by 90 days and does not have an outcome of death or transferred to another clinic [1, 10]. A patient who is not lost to follow up, and doesn’t have an outcome of death or transfer, is considered retained in care.

Patient engagement is increasingly recognized as an integral part of health care and a critical component of patient-centred services where a patient actively chooses to take part in decision making about their care options with their healthcare provider(s) [2, 12, 13]. In addition, clinical and health care resources may be better used if they are aligned with patients’ needs which is critical for the sustainability of health systems worldwide [13]. Re-engagement in care is when the patient actively chooses to return to care after previous disengagement. Re-engagement in care is when a client has made a health facility visit after being lost to follow up [14, 15]. These ancillary services can help patients navigate lifelong treatment.

Research on the reasons people disengage from care has identified individual factors such as fear, or unstable income; social and interpersonal factors ranging from disclosure and the treatment itself; and clinical factors including the provider attitudes and structural issues.

Individual factors include forgetting to take medicine, negative attitudes towards healthcare, and common mental health disorders like depression which could exist prior to or after the HIV diagnosis [9, 10, 16–18]. Being ill is associated with disengagement from care, perhaps in part because clinic visits begin to become physically unmanageable as the severity of an illness progresses [16]. Issues of distance and travel time also compound the disruption that accessing ART can have on daily tasks; people are busy and are subject to multiple demands on their time including work and family duties which can prevent regular access to care [2, 5, 17].

At the interpersonal and social levels, stigma and lack of familial or peer support can be major factors, making access to care challenging [2, 6]. The passive withdrawal or lack of typical familial and peer support structures can be detrimental and contribute towards disengagement [8, 9]. In low resource areas in particular, this type of support can be extremely important as family and friends serve a multitude of functions that the more affluent could pay for, in example, supervising children while a care giver is at the clinic or providing transportation.

Research demonstrates the importance of patient-provider relationships to initiating and maintaining ART [2, 5–9, 11, 14, 19]. Care experiences at clinics are affected by all staff including receptionists, testing staff, nurses and doctors [6–9, 15, 20–23]. Trust is key to building good interpersonal relationships, however high turnover rates make fostering trust difficult, negatively affecting care engagement [2]. Ensuring retention can also be difficult when there are insufficient numbers of skilled and trained staff to provide good quality of care [2]. Lack of empathy, bias or disrespect on the part of clinic staff, can also be key factors in disrupting care; many clinic-attendees speak about being treated unkindly by staff who may shout at or otherwise subject them to rough treatment [7–9, 15, 20–23]. Finally, coming back to a clinic after a break in treatment can be overwhelming, and patients might anticipate or actually experience negative reactions from clinic staff; this serves as further reason to remain disengaged from care [8, 9].

Accessibility also plays a key role in keeping patients engaged in care; this includes clinic operating hours and where these sites are placed in relation to residences or ‘catchment areas’ [2, 6, 9, 20, 24, 25]. Those clinics where workflow is not streamlined and where there are long delays or wait times [2] for appointments, or challenges with appointment-scheduling [18] are also susceptible to higher rates of disengagement with care.

Research has indicated that there is a need to pay particular attention to groups at heightened risk including: 1) stigmatized groups such as female sex workers, the previously incarcerated, people who abuse alcohol or other substances, transgendered people; 2) those from resource-scarce backgrounds like people with lower levels of education, people living in rural areas, people with unstable or uncertain access to housing, people who are mobile and those who have relocated, and those who currently have limited access to resources [3]; 3) men, particularly younger men, who are at risk of either not entering, or of falling out of the care cascade at multiple points; and 4) postpartum women, who after completing PMTCT programs and successfully preventing transmission to their infants, often drop out of care and, 5) adolescents and young people [1, 2, 16, 25, 26].

### The welcome back campaign intervention

Although South Africa is close to achieving the first 90 target of all PLHIV knowing their HIV status, the country is falling short on the second 90, with only 70% of those with known status on ART [22]. Improved retention in care is needed to get closer to sustained ART for all. In January 2019, Anova Health Institute conducted a campaign for fifteen months to encourage ART patients who had interrupted treatment to return to care. The campaign consisted of training for health care workers, using training modules developed by Médecins Sans Frontiers (MSF) and mass media messaging. As part of an evaluation of the campaign, we interviewed patients returning to care in order to understand barriers and facilitators to retention in primary care public-sector clinics in Johannesburg, South Africa. The aim of this study was to investigate the experiences of patients returning to care after interrupting treatment to better understand why they disengaged and returned to care, in order to align services better with patients’ needs.

## Methods

### Study site

Our study was conducted in one region of Johannesburg, Region E, which includes Alexandra township [2]. * Region E makes up about 14% of the Johannesburg population of approximately 700,000 people [23]. There are nine clinics, including one community health centre that provides a wider range of services. A total of 31,714 HIV patients in April 30, 2020 access ART at these nine clinics.

### Ethics approval

Ethical approval for the study was granted by the Human Sciences Research Council, Research Ethics Committee (HREC) (approval HREC Number: REC 3/22/08/18). Written consent was obtained for interviews.

### Study design

This mixed methods study consisted of two components. 1) Data collected from all patients re-initiating ART was entered into a structured tool by their healthcare providers at the nine participating clinics from July 2019 to March 2020, 2) We conducted semi-structured interviews with a sub-set of these patients.

### Quantitative

The quantitative component captured numerically of responses for reasons of; disengagement from care; returning to care and; remaining in care. Data was captured for a total of 562 people re-initiating ART between July 2019 and March 2020 (9 months).

#### Instrument: Patient re-initiation tool

Information was collected by health providers for all patients re-initiating ART to facilitate appropriate counselling and adherence planning. A patient re-initiation survey tool was used where participants could give counsellors multiple responses in which multiple options could be checked boxes could be completed for questions. This data was captured anonymously into a REDCap (Research Electronic Data Capture) database [27] to investigate reasons for disengagement (including interruption of ART) and returning to care.

#### Recruitment and sampling

Study participants were recruited from all nine clinics, aiming to achieve representation of men and women, as well as different age groups. All patients who either a) self-reported they were re-initiating ART or b) were identified when the patient tested positive for HIV and were found to already appear on TIER.net, a national monitoring system used that captures patient-level HIV information at health facilities [28]. All participants had to be 18 years or older were eligible for inclusion. Counsellors invited all patients re-initiating ART to participate in the study on a voluntary basis.

#### Data collection and management

The tool had both check box answers and free text responses which included reasons for disengagement from care, why they decided to come back to the clinic, why they chose the specific clinic they were attending, and how they would like to be supported to stay in care.

#### Analysis

A descriptive analysis was conducted of the survey responses. We report frequencies and percentages for each response.

### Qualitative

The qualitative component aimed to investigate patient perceptions to understand the reasons for disengagement from care in more depth, including contextual factors that could not be fully explored in the surveys. A total of 30 IDIs were completed across the nine clinics.

#### Instrument: Semi-structured interview guide

An interview guide was used that covered participants’ experiences testing for HIV, thoughts and feelings about taking ART, other treatment (i.e. traditional/conventional medicine), and any ongoing challenges which might cause disengagement from care.

#### Recruitment and sampling

Study participants were recruited from all nine clinics, aiming to achieve representation of men and women, as well as different age groups. All patients who were re-initiating ART and 18 years or older were eligible for inclusion. Counsellors invited all patients re-initiating ART to participate in the study on a voluntary basis, the names and contact details of those who agreed were shared with the research team. In depth interviews (IDIs) were completed in person or telephonically.

#### Data collection and management

With permission, the interviews were audio recorded and transcribed with simultaneous translation into English. Languages included isiZulu, SeSotho, SeTswana and English. Transcripts, participants’ demographic information, and journal memos including observations of participant behaviour were entered into NVivo 12.0 (QSR International Pty Ltd., 2018). Reflective journaling was used to enhance authenticity of the research process and findings by understanding any preconceived ideas from the research team. Memos were used to maintain a process log and capture any observations especially when conducting the interviews. Memos and journals allowed the research team to maintain a record of analytic decisions throughout data collection and analysis.

#### Analysis

A thematic approach was used to investigate the experiences of patients who re-initiated ART [29].

The process of analysis began with open coding directly from the data. Codes were grouped into categories of reasons for disengagement from care. The fourth version of the coding scheme reached consensus amongst four researchers in the team. The categories were reduced to identify key themes using the conceptual framework discussed below. Any commonalities and differences between high risk groups were noted.

### Conceptual framework

The McIntyre, et al. framework for understanding access to healthcare was used in the development of themes, and the reporting of findings. Access is defined as having three dimensions: availability; affordability and acceptability [30]. Availability speaks to whether HCWs and HIV services are supplied in an appropriate manner. Affordability examines the patients’ ability and willingness to pay in order to receive and access healthcare services [30]. This is not just monetary but also measured as opportunity costs of whether patients can or cannot afford to take time off work to get to the clinic [30]. Acceptability explores the relationship between the healthcare provider and patient including expectations of each other, perceptions towards characteristics such as age, gender, language which can impact the delivery and recipient of care [30].

## Results

### Re-initiation tool

We captured data for a total of 562 people re-initiating ART between July 2019 and March 2020 (9 months), 66% of whom were women (Table 1).

**Table 1:** Demographics and characteristics: Patient re-initiation surveys.

Variable	Sub-category	Frequency	Prevalence (95% CI)
Age years (554)	15–24	46	8% (6 to 11)
	25–49	417	75% (71 to 79)
	50+	91	16% (14 to 20)
Gender (489)	Woman	323	66% (62 to 70)
	Man	158	32% (28 to 37)
	Transgender	8	2% (1 to 3)
Previous ART Clinic (538)	Returned to same clinic	396	74% (70 to 77)
	Moved to another clinic	142	26% (23 to 30)
Location of previous ART clinic if moved (141)	In Johannesburg	85	60% (52 to 68)
	Outside of Johannesburg	10	7% (4 to 13)
	Other province in South Africa	36	26% (19 to 33)
	Outside country	10	7% (4 to 13)
Patient time off treatment (515)	1–3 months	112	22% (18 to 26)
	4–6 months	139	27% (23 to 31)
	7–12 months	101	20% (16 to 23)
	More than 12 months	163	32% (28 to 36)
Missed medication frequency prior to treatment interruption (434)	Almost never missed	327	75% (71 to 79)
	Missed <1 a month	36	8% (6 to 11)
	Missed >1 a month	48	11% (8 to 14)
	Missed > 1 a week	23	5% (4 to 8)

### Reasons for stopping ART

**Fig 1:**
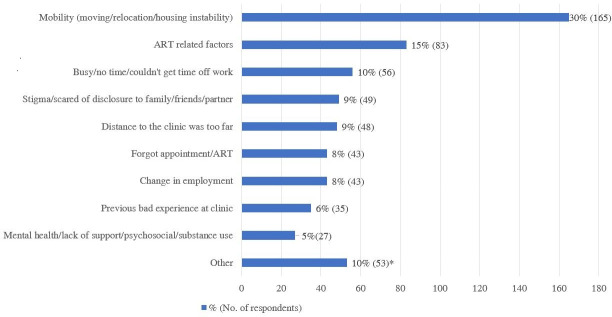
Reasons for stopping ART % (No. of respondents). Other reasons for stopping ART included: 13 (2%) with no significant reason, 11 (2%) incarceration, 8 (1%) hospitalised, 8 (1%) preferred faith based healing or traditional healers, 6 (1%) had insufficient food, 3 (1%) stopped with following birth of a child. ^a^ Participants could choose multiple responses.

The three most common reasons reported by patients for disengagement from care were mobility (165 patients, 30%), such as moving, relocation or housing instability; ART related factors such as side effects or pill burden (83 patients, 15%); and time limitations (56 patients, 10%) such as not being able to take time from work to get to the clinic (Fig 1).

### Reasons for returning to care

**Fig 2:**
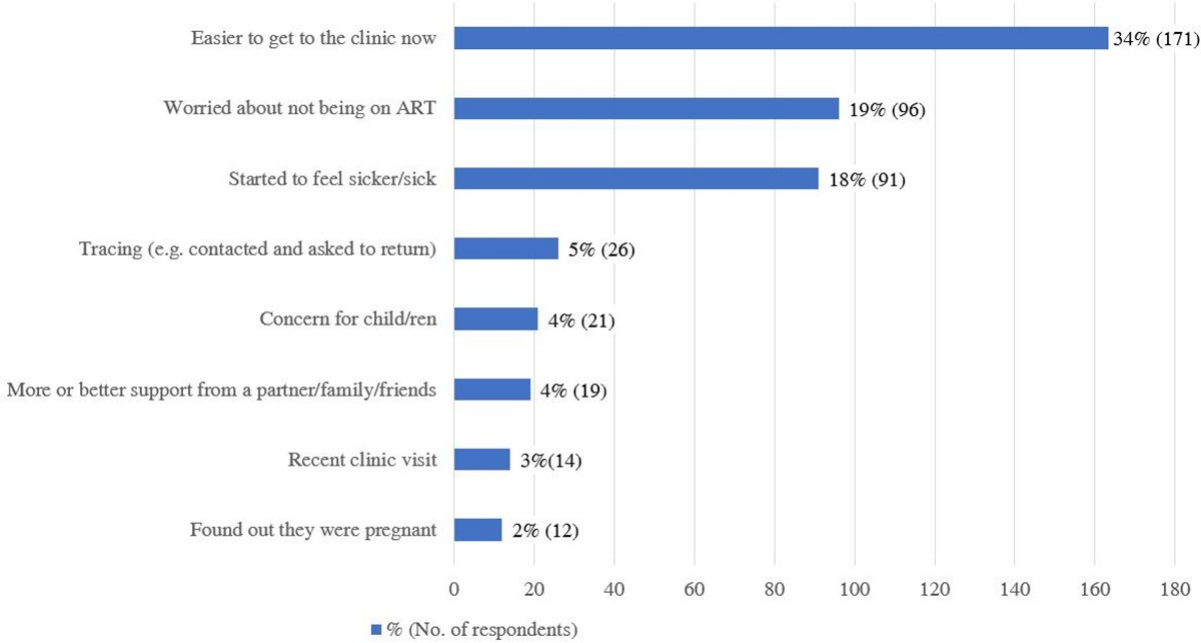
Reasons for returning to care % (No. of respondents). ^a^ Participants could choose multiple responses.

171 (34%) patients reported it was easier for them to get to the clinic due to a change in circumstances, followed by 96 (19%) who were worried about not being on ART and 91 (18%) who were feeling sicker (Fig 2). 26 (5%) patients returned to care due to tracing (contacted and asked to return by clinic staff). Other reasons included concern for child/ren (21, 4%), more/better support from partner/family/friends (19, 4%), recent clinic visit (14, 3%), and finding out they are pregnant (12, 2%).

Furthermore, when patients were asked why they chose the clinic they visited, 386 (79%) said they chose the clinic nearest to where they live, followed by 30 (6%) who chose the clinic nearest to their work.

### Factors influencing patients to remain in care

When patients were asked what support could help them remain in care, 170 (36%) patients requested short messaging services (SMS) reminders, followed by 135 patients (29%) requested counselling, 65 (14%) patients wanted shorter queues and 38 (8%) wanted check-in phone calls.

### Semi-structured interviews

*Demographics & characteristics*. A total of 30 semi-structured interviews were completed (Table 2).

**Table 2:** Demographics of participants.

Variable (number of responses)		Frequency (%)
Age years (30)	15–24	5 (17%)
	25–49	24 (80%)
	50+	1 (3%)
Gender (30)	Woman	19 (63%)
	Man	11 (37%)
	Transgender	0 (0%)
Occupation (28)	Unemployed	18 (64%)
	Formal employment	7 (25%)
	Informal employment	2 (7%)
	Self-employed	1 (4%)
Highest level of education (30)	Primary school	3 (10%)
	Secondary high school	26 (87%)
	University	1 (3%)
Marital status (30)	Single or never married	18 (60%)
	Married/cohabitating	10 (33%)
	Divorced	2 (7%)

Findings have been organised into health service and patient factors. Health service findings are reported based on the Accessibility framework and its three components; availability; affordability and acceptability.

### Healthcare services

#### Availabilit*y*

Services that extended beyond clinical visits facilitated retention in care. Participants reported that monthly SMS treatment reminders and having the clinic staff phone them for follow-up appointments or check-ins had influenced their return to care. Participants “felt welcomed and open” [IDI 19] and the phone calls from the counsellors were well-received,

*“I felt good because sometimes when you get that call from the clinic*, *the first thing that comes to mind is -what were you doing when you stopped taking treatment*, *those kinds of things but I didn’t get that”* [IDI 4, Female, 34 years old].

Available services were not always of a high enough quality to encourage retention, particularly when side effects were experienced. When side effects were not adequately addressed by providers, it led to disengagement from care. A participant explained that health talk sessions could serve as a platform to help encourage other patients to stay on treatment, no matter the side effects that someone is experiencing,

*“If we can have a session where we are asked how do we feel about taking treatment*. *It’s important just to come and sit*, *[because] maybe someone is having some side effects*. *There lies the problem*. *Maybe [some]one when they take treatment*, *they have dizzy spells and this one doesn’t have those dizzy spells*. *This one with dizzy spells should be encouraged by the one who doesn’t have dizzy spells but if he or she today drinks and tomorrow doesn’t drink because of those dizzy dreams then [they] will end up leaving the treatment*, *but if we encourage- I think the best thing is to strengthen the health talks*.*”* [IDI 13, Male, 41 years old]

Furthermore, availability of clinical services was limited by high patient loads and long queues which posed as barriers particularly for men. A 47 year old male stated,

*“you find that you get here at six in the morning and you end up leaving here at three in the afternoon without getting the treatment because they have turned you away and then you have to come back the following day and you know how we are as people*. *We would give up on taking treatment because of waiting for that long”* [IDI 18].

When interviewing patients about their duration off ART, seven patients responded they were off treatment for more than one year,

*“I kept telling myself that I have to come back and then until I told myself that I am stopping and it was almost year I think it was around May or June when I decided to stop*. *I only took those pills for one month and after that*, *I didn’t continue”* [IDI 15].

Patients expressed that they were taking another persons’ treatment. A 46 year old female patient states, “*I was taking my sister’s treatment*. *Even then I was not taking them regularly because I didn’t want her to run out of them before time so I will skip 2 days without taking them*.*”* [IDI 19].

Stock shortages were also a concern and were reported by participants to be a likely reason for them to discontinue treatment in the future. A 41 year old male expressed that he will go extreme lengths to have his ART, *“Unless if there is no treatment but because I know the importance of taking treatment if I can’t find it here then I would try and look for it somewhere else”* [IDI 13].

Services were made unavailable to patients who were highly mobile because of the bureaucratic barrier of requiring a transfer letter. The requirement for a transfer letter when moving between clinics was a common reason for disengaging from care. Highly mobile populations who may not be able to predict or control when or where they move, are often required to keep a handheld transfer letter which results in a common reason for disengaging in care, especially when a patient has to manage the paperwork when moving around. A 29 year old, post-partum woman states that she moved from KwaZulu-Natal province in 2017 to Johannesburg but that she did not disengage from care until there was miscommunication about transfer letters and treatment access with the clinic.

*“I took a transfer letter and went back home and I had to come back to this side and then I left the file* [transfer letter] *that side telling myself that I will go back home […] I came back here because the treatment was finished*.*”* [IDI 1]

Mobile participants often forgot their medical documents such as transfer letters for attending a different clinic for ART, or appointment cards in their home country and needed monthly reminders about their HIV treatment and care. Participants expressed that managing their ART was difficult when moving between countries or provinces, especially with the barrier of a transfer letter to gain access to health care services. On the other hand, clinics that had flexible and extended hours facilitated keeping these participants in care. *“I chose this [24 hour] clinic because it is closer to where I stay and it doesn’t close*.*”* [IDI 18]

In addition, the clinic location and having it be closer to home was essential to remaining in care for those that were mobile. Clinics that had flexible and extended hours were a very influential in keeping these patients in care.

#### Affordability

Many participants endure opportunity costs when it comes to accessing services and ART. Older male participants in particular expressed that they could not afford to take time off from work,

“*It’s closer to my workplace because I work here in Sandton*, *it will be easy for me to come and get them unlike if I am working far then I have to ask for leave and then they will ask a whole lot of questions and you know the stigma that comes with it*, *so*, *in my lunchtime*, *I can come and collect and go back to work”* [41 year old male, IDI 13].

Participants reported coming back into care when it became manageable for them, including when they were closer to clinics and transport costs became more affordable. However, others with continuing transport challenges only returned to care to restart ART due to becoming sick, weak, and losing weight.

Employment stability was seen as a facilitator of retention. A 34 year old female stated when asked how she could be supported to remain on treatment, *“The only thing I want is to find employment so that I can be able to support my kids […] because it can give you more and you can buy food as well”* [IDI 15].

#### Acceptability

Administrative difficulties, such as the loss of medical records, as well as the attitudes of clinic staff led to frustration in participants.

*“Apparently I had to go to the computer and there were some complications and so when I got there I think my form was not there and then there were some misunderstanding they told me that maybe I didn’t bring it or whatsoever and then I just got pissed off and then I decided that it’s better if I stopped”* [IDI 4].

Complicated procedures, and poor communication of these, also led to participants giving up, and not returning to care, a 47 year old male explains,

*“They told me that they would have to reschedule my appointment*. *I did explain to them that I tried coming here and they gave me another number and they said when I come next time I should show them and when you come the following day they tell you a different story”* [IDI 18].

Participants also reported stopping treatment due to the lack of support systems and inability to rely on a support structure when undergoing challenges with their health.

Health service providers can help to fill the gap with supportive care, however, participants reported disengagement from care when they did not feel supported by health staff and the services provided. For example, a highly mobile and stigmatised group were the previously incarcerated. Findings revealed a total of eleven participants in the study had been formerly incarcerated which caused them to stop ART. Previously incarcerated individuals are often not well received at clinics following their release. Staff bias and negative attitudes can impact on engagement and contribute to fear and judgement for the individual. A 33 year old male expressed how he lost his job and ended up incarcerated where he got ARVs in prison from another inmate. He explained,

*“I told him that I am positive […] and I can’t get home at the moment*. *My sister use to go and collect treatment for me while I was at work so now I can’t get my treatment*. *This guy said to me that he has plenty of these pills*. *The thing is I was afraid of telling the nurses there that I am positive*. *This guy gave me one container*[..] *I think about my life*. *If I take it [ART] for 5 years maybe it would give me 5 more years again*” [IDI 11].

This vulnerable population who may want to return to care to prolong their life and are afraid to fall sick, struggle due to possible judgement and fear that act as deterrents in returning to the clinic.

Male participants struggled to find a balance between their health needs and managing their responsibilities at work and at home. Older men indicated that severe health issues such as cardiac issues (i.e. heart attack) or surgery, interrupted their ability to stay on ART. Some men expressed reasons for lack of engagement in this type of care often due to men feeling excluded and disapproved of in clinic settings. However, men felt that more support is needed where they could interact with their peers which could be used as a way to better cope with managing ART. A 41 year old male participant stated, *“Support groups will be helpful and meeting other people who are also HIV positive so that we can encourage each other”* [IDI 15].

On the positive side, the importance of networks and finding social support in clinics was commented on by many participants. A 41 year old male expressed that, *“encouraging my peers and being able to communicate with others in clinic health talks is the way I can network and build support in the clinic*. *I may find out they share very similar or different experiences”* [IDI 20].

Such positive interactions help to build capacity of a patient and social resources, preventing disengagement from care. Other reasons for remaining in care included perceptions of staff competence, and improved relationships with staff.

### Individual factors

Some participants had needed time to digest their diagnosis and its consequences, and only then did they start to feel that being on treatment was the best way to look after their health. A 34 year old male stated, *“This thing [treatment] is always on my mind and it keeps coming back that I have to attend to it*.*”* [IDI 15]. Many were pushed to return to care due to feeling increasingly unwell and experiencing visibly poorer health (for example weight loss).

Some participants had discussions with partners, family members and friends in their support network before coming back to the clinic to re-start treatment. These often included individuals going through similar experiences and the networks became a source of guidance. A 37-year old female stated, *“I discussed it with my boyfriend*. *I told him that he cannot take treatment alone and then he encouraged me to go and start treatment again”* [IDI 30].

Participants were asked about any ongoing challenges that they were experiencing that could lead to further disengagement from care. The dominant view was that they do not see themselves interrupting treatment again. However, inadequate food supply and lack of employment were prominent concerns that patients spoke about; A participant stated when asked what can we do to help remain on treatment, *“The only thing I want is to find employment so that I can be able to support my kids […] because it can give you more and you can buy food as well”* [IDI 15].

Often participants have support networks that include individuals going through similar experiences like themselves with ART. A participant expressed him and his wife built a support network after finding out they were both HIV positive, using their children as motivation for adherence to ART, *“I felt like my life was ending because I didn’t know where I got it*, *and it’s not as if I have multiple partners you know*. *I told myself that I need to go forward with my life and then I told my wife to go and check and she found out that she is also positive*, *so taking treatment was [from] the pressure of protecting the children”* [IDI 25].

These networks can become a source of guidance on a personal and emotional level that can encourage participants to stay on treatment. Participants’ reliance on a strong support system while they endure many difficulties in achieving stability on their ART is important. Participants were not able to receive proper healthcare support in one clinic alone which then contributed to mismanagement of their adherence to treatment due to constantly moving to different clinics whether in or outside Johannesburg.

## Discussion

Changing life circumstances was the most prominent reason for disengagement from care in our study participants, in both the return to care surveys, and the qualitative analysis. Services were made unavailable to patients who were highly mobile, for example by requiring a transfer letter, a which became an important barrier to engagement in ART care. Almost a third of patients returning to care reported that mobility was the main cause for their disengagement from care with an additional 10% unable to take any time off work; conversely more than a third returned to ART due to improved circumstances. Health services were not perceived to be responsive to life changes or mobility, leading to disengagement from care.

Gauteng Province has the most mobile population in South Africa with both internal and cross-border migration occurring with those who move from province to province or from another country seeking healthcare access or better livelihoods [31, 32]. Lack of health system responsiveness to mobile people is likely to have a large impact on retention in the province, and throughout the country. As demonstrated by the findings of this research in Johannesburg, patients who are highly mobile find it very difficult to remain engaged in care and adherent to ART [3–5, 17, 25, 26]. Ensuring full implementation of a unique identifier and a nationally linked database where an individuals’ ART history can be accessed could help resolve the issue of transfer letters and loss of healthcare documentation [33]. A card or barcode could also be linked to records to maintain continuity of care. Individuals that are highly mobile and moving between facilities should also be advised to give their appointment cards to a trustworthy person and provide this persons’ contact number to clinics. However, work needs to be undertaken to educate all facility staff about the importance of maintaining an individual’ access to treatment even if a transfer letter is unavailable [33].

Similar to the findings of other research [14, 16, 21, 26, 30, 33], barriers related to acceptability included staff attitudes, waiting times and difficult to understand procedures, highlighting the importance of positive care experiences and trust between the service provider and service user [2]. Interventions are needed to shift attitudes and norms towards being more accepting of re-engaging clients. These include values clarification workshops for both healthcare providers and patients to help develop a mutual understanding between the service provider and user; friendly health talks and information, education and communication (IEC) materials catered to specific patient populations; and training for healthcare providers to reinforce positive messaging and to prevent stigmatisation, including avoiding labelling individuals with the term “defaulter”.

Affordability and competing responsibilities were also important to access, particularly related to transport costs and opportunity costs relating to missing work, with most clients attending the clinic closest to where they live/work. Though HIV differentiated services are available to support long-term adherence by simplifying and spacing treatment collection with options closer to home, patients’ awareness of these options remains limited [12]. More efforts are needed to inform the community at large about the activity that has been undertaken to ensure stable clients can access continuous medication as easily and as efficiently as possible. Provision of flexible/after hour services and community-based services are critical to improving access.

Commonly requested support interventions including SMS reminders, counselling, and proactive check-in phone calls should be provided to facilitate retention. Support groups were also identified by participants as an important resource to keep them in care. Participants appreciated the opportunity to network and learn from peers who are experiencing the same treatment journey as they are. It could prove beneficial for the HIV programme in South Africa to make more extensive use of peer educators and peer navigators, who are themselves expert patients, to engage and support those who are at greatest risk of disengaging from care.

Literature suggests that multiple individual factors can intersect to lead to increased risk of disengagement [10] in our participants [2], for example post-partum women who were highly mobile or amongst eleven formerly incarcerated men who were unable to afford transport costs. Though only 2% of study participants disclosed they were previously incarcerated, it is important to recognize the power each individual story holds to understand more deeply what challenges exist around retention. It is important that we understand these are not mutually exclusive groups, and the same individual can be in more than one group, and in some instances, more than one concurrently, during the life course of HIV [2, 12, 32]. Patients need to be understood not just in terms of their demographic profile, but also with a deeper understanding of their social circumstances.

Unfortunately, many participants only returned to care when they felt sicker which has negative effects on their short and longer term outcomes. Health services need to provide more accessible services [13], by improving service acceptability, to encourage earlier return to care and improved individual and population outcomes.

### Strengths and limitations

This study has combined quantitative and qualitative data to develop an in-depth understanding of why people dropped out of and returned to care.

Social desirability bias may be a limitation, since service providers assisted with the patient re-initiation surveys, and patients may have avoided complaining about negative experiences. People returning to care are not representative of those who remain disengaged, and we cannot know how their experiences may differ from those still absent from care.

## Conclusion

While there are many barriers to remaining in care, fortunately there are also a number of ways that engagement can be improved and the likelihood of remaining on ART can be increased. This means that while health services cannot necessarily ensure that everyone will remain in care, they can make it decidedly easier for patients to do so, and for them to return to care should they temporarily disengage. This study confirms previous research that more client-centred and responsive health services should improve retention on ART. The healthcare system needs to support, and actively listen to, patients, to reduce risk of future disengagement from care, including those who are experiencing difficult or changing life circumstances.

## Supporting information

S1 File Qualitative interview guide. (DOCX)

S2 File Anonymized transcripts. (PDF)

S1 Dataset Anonymized quantitative data set. (XLSX)
