# Supplementary material for: Why do patients interrupt and return to antiretroviral therapy? Retention in HIV care from the patient’s perspective in Johannesburg, South Africa
Source: PLoS One. 2021 Sep 2;16(9):e0256540. doi: 10.1371/journal.pone.0256540 (PMC8412245; doi:10.1371/journal.pone.0256540)
Supplement: S1 File — (DOCX) [file pone.0256540.s001.docx]

F.4 Patient Interview Guide

**IN DEPTH INTERVIEW GUIDE**

**Introduction**

My name is ----------------------------------------------------(Name of Interviewer) working on a research project supported by Anova Health Institute. We are conducting research on our programme supporting the government in providing HIV and TB treatment and prevention services and its impact on health services and the community. We are conducting a study to find out about how the Welcome Back Campaign is being implemented and whether it is making a difference in returning clients to care, like yourself and in the work of health workers and their community. It is important for us to collect this information in order to improve our programme, and the support we provide to government health services.

**Demographic Information**

| **1** | **Age (in completed years)** |  |
| --- | --- | --- |
| **2** | **Gender** | 1.  Man  2.  Woman  3.  Transgender |
| **3** | **Marital Status** | 1.  Single or never married  2.  Married  3.  Married but separated  4.  Cohabiting  5.  Divorced  6.  Widowed |
| **4** | **What is the highest level of education completed?** | 1.  Primary school education  2.  Secondary high school  3.  College/vocational  4.  University  5.  Literacy classes only  6.  Other (specify---------------------------------------- |
| **5** | **Employment Status** | 1.  Not employed  2.  Employed, formal  3.  Employed, informal  4.  Self-employed |

1. What service(s) have you come for today?
2. Why did you choose this facility? (***Probe: convenient times, staff attitudes, location, waiting time)***

**Reasons for stopping and restarting**

1. How long would you say you were off HIV treatment before visiting the facility? **(*Probe: duration: 1-6 months, 7-12 months, more than 1 year)***
2. What treatment were you taking? ***(Probe: type of treatment – conventional medicine, traditional, religious/spiritual)***
3. Why did you interrupt/stop your treatment? ***(Probe: moving/relocation, housing instability, couldn’t get time off work, ART side effects, start to feel better/too worried)***
4. Did you struggle to take the treatment every day before you stopped? ***(Probe: missed doses)***
5. Is this the first time you stopped your treatment, or have there been other times?
6. Why did you decide to return to care? ***(Probe: reasons, triggers, was this a difficult decision)***
7. Did you discuss your decision with anyone before coming to re-start treatment? ***(Probe: why? who?)***

**Experiences with/ Attitudes to ART**

1. What was your experience when you tested for HIV and were first diagnosed? ***(Probe: staff attitudes, privacy and confidentiality concerns, unpleasant, why was it so?)***
2. What are your thoughts about taking HIV treatment? ***(Probe: cultural, power differences, identity, meaning and importance patients attribute to adherence, any changes in understanding over the course of treatment).***
3. Is taking HIV treatment important to you? Why? (***What would happen if didn’t take treatment every day?)***
4. Have you gotten treatment at any other clinic/hospital before? Where? Why? What was your experience? Who assisted you at the facility? ***(Probe: CLO, Nurse, Admin Clerk)***
5. Have you taken any other treatment for HIV before? ***(Probe: type of treatment – conventional medicine, traditional, religious/spiritual)***
6. If you knew someone with HIV, would you recommend that they take treatment? What else would you recommend they do to stay healthy?
7. Now that you are back, are there any ongoing challenges you have which might make you stop treatment again or might prevent you from staying in care? Please explain. ***(Probe: stigma and discrimination, interference with daily schedules, side effects, staff attitudes, costs, fear of disclosure, food insecurity, lack of social support***
8. What could be done to help you remain on treatment and stay in care? ***(Probe: staff attitudes, health systems)***

**Experience restarting**

1. How do you feel about your clinic visit today? **(Probe: waiting time, were staff friendly)**
2. Was your visit today better/ worse/ the same as previous visits?
3. What do you like most about this facility?
4. What do you like least about this facility?
5. If you could change anything about your experience of restarting treatment, what would you change?

**Welcome Back Campaign**

1. Have you heard anything on the radio, or seen any adverts about coming back into care? Or about your clinic?
2. Have you ever been phoned by the clinic? How did you feel about being phoned?
3. Did anyone visit you at your home and encourage you to come back to the clinic?
4. Have you heard of the Welcome Back Campaign?
5. If yes to any of the above, did the adverts or phone calls influence your decision to restart treatment?
