# Supplementary material for: Why do patients interrupt and return to antiretroviral therapy? Retention in HIV care from the patient’s perspective in Johannesburg, South Africa
Source: PLoS One. 2021 Sep 2;16(9):e0256540. doi: 10.1371/journal.pone.0256540 (PMC8412245; doi:10.1371/journal.pone.0256540)
Supplement: S2 File — (PDF) [file pone.0256540.s002.pdf]

Interview Type: IDI

SITE: Clinic B

INTERVIEWER: FB

TRANSCRIBER: FB

TRANSLATOR: FB

DATE OF INTERVIEW: 06/03/2020

DATE OF TRANSCRIPTION: 10/03/2020

TIME: 20:59

INTERVIEWER: Okay, thank you once again for your time. Like I said earlier what I am going to do is just to ask you a few questions about the services you received from the clinic today. Basically what we are doing is to offer support to the government in the service of providing HIV and TB treatment. So, we are conducting a study to find out how is the welcome back campaign is being implemented here and is it making a difference to clients who are returning like yourself welcome back campaign is welcoming you back to treatment, so, this about you and if it happens while we are talking that you mention your name when I write this down I won't include your name when I write this down. So, just to understand again for record purposes can you please tell me your age?

RESPONDENT: I am 29 years.

INTERVIEWER: 29 years. Okay, are you a male or female?

RESPONDENT: Female.

INTERVIEWER: Okay, are you married or not?

RESPONDENT: I am single

INTERVIEWER: Are you in a relationship though?

RESPONDENT: No.

INTERVIEWER: Okay, what is your highest level of education?

RESPONDENT: Grade 12

INTERVIEWER: Okay, what is your employment status?

RESPONDENT: I am unemployed.

INTERVIEWER: Okay, the first question is what service did you come here for today?

RESPONDENT: They called me and said I must come for an interview with ANOVA.

INTERVIEWER: Okay, besides that?

RESPONDENT: That is the only thing.

INTERVIEWER: So, you came for the interview and nothing else like for treatment?

RESPONDENT: No, I was here for treatment yesterday.

INTERVIEWER: Okay, we will look at yesterday's visit, why did you choose this clinic specifically, 8th avenue?

RESPONDENT: It's because that is where I stay.

INTERVIEWER: So, you stay here on 8th avenue?

RESPONDENT: I stay at 12, it's around here.

INTERVIEWER: So, you choose this clinic because you stay around here?

RESPONDENT: Yes.

INTERVIEWER: And then in terms of staff attitudes, how are they? Apart from choosing that you stay here, how are they?

RESPONDENT: They are fine.

INTERVIEWER: you don't have any issues with them?

RESPONDENT: No.

INTERVIEWER: And what about waiting time?

RESPONDENT: In terms of waiting time, we wait here but in the end, they do help us.

INTERVIEWER: How long do you have to wait?

RESPONDENT: If you get here around 8 in the morning you can leave the clinic around 10 to 11 in the morning.

INTERVIEWER: So, that time for you it's fine?

RESPONDENT: It's the one they are using.

INTERVIEWER: Okay, thank you for that. Okay, some of the questions I am going to about treatment, firstly, have you ever stopped taking treatment before?

RESPONDENT: Yes.

INTERVIEWER: I am asking this because the following questions are about stopping treatment and starting treatment. This question is asking, the time you stopped treatment, for how long did you stop taking treatment?

RESPONDENT: I took a transfer letter and went back home and I had to come back this side and then I left the file that side telling myself that I will go back home and that is when I didn't go back home and I came back here because the treatment was finished.

INTERVIEWER: When did you go home?

RESPONDENT: In December but I took the transfer in 2014

INTERVIEWER: So, you took the transfer letter in 2014 and went home?

RESPONDENT: Yes, and when I came back here I forgot the file at home.

INTERVIEWER: And when did you come back here?

RESPONDENT: I came back on the 24.

INTERVIEWER: 24 this year?

RESPONDENT: Yes.

INTERVIEWER: 24 which month?

RESPONDENT: 24 February.

INTERVIEWER: When did the treatment run out?

RESPONDENT: When I came back here.

INTERVIEWER: Let me say, how long have you not been taking treatment?

RESPONDENT: It's been 3 months.

INTERVIEWER: It's been three months since you took your treatment, in these three months is there anything that you have been taking?

RESPONDENT: I wasn't drinking anything.

INTERVIEWER: Is there anything that you were doing or you were not taking anything?

RESPONDENT: I wasn't doing anything.

INTERVIEWER: You weren't taking any traditional medicine or going to church or anything like that?

RESPONDENT: No.

INTERVIEWER: What made you stop taking treatment for those 3 months?

RESPONDENT: I was waiting for my file form that side and they said they want me and I can't send someone.

INTERVIEWER: Okay, where is home so that we can understand?

RESPONDENT: Limpopo.

INTERVIEWER: So, that side they want you?

RESPONDENT: Yes, because I have to give them a file number because I left it that side.

INTERVIEWER: Okay, is that the only reason that made you top taking treatment?

RESPONDENT: Yes.

INTERVIEWER: Okay, before you stop taking treatment, has it happened that you struggle with taking treatment then or you were taking treatment all the time and correctly?

RESPONDENT: I was taking it correctly.

INTERVIEWER: So, you didn't have any issues about taking treatment?

RESPONDENT: No.

INTERVIEWER: Okay, and then you have never missed taking treatment before the 3 months?

RESPONDENT: Yes.

INTERVIEWER: Was this your first time stopping to taking treatment or has it happened before?

RESPONDENT: It was the first time.

INTERVIEWER: And then, what made you want to return to care after the 3 months?

RESPONDENT: I was starting to get sick.

INTERVIEWER: You were starting to get sick?

RESPONDENT: Yes. I had developed a rash on my skin and then I had to go to the clinic and start from the beginning.

INTERVIEWER: Okay, starting to be sick is the reason that made you come back?

RESPONDENT: Yes.

INTERVIEWER: Coming back to start treatment again did you discuss it with anyone, like telling someone that you want to go back to treatment?

RESPONDENT: No.

INTERVIEWER: You just decided to come back by yourself?

RESPONDENT: Yes.

INTERVIEWER: Okay, can we go back a little bit, the first time you tested and found out that you positive, your experience how was it?

RESPONDENT: I didn't believe it but I told myself that I have to accept it.

INTERVIEWER: Did you have any issues in terms of privacy, confidentiality or you didn't have any issues at all?

RESPONDENT: No.

INTERVIEWER: You didn’t have any issues?

RESPONDENT: No.

INTERVIEWER: In terms of staff attitudes where you were testing, how was it?

RESPONDENT: The explained everything to me everything I should do and also how I should live my life going forward, they told me everything.

INTERVIEWER: So, you are happy with the staff attitude of that place?

RESPONDENT: Yes.

INTERVIEWER: What are your thoughts when it comes to taking treatment? I sit a good thing or bad thing or do you think there is another way of treating HIV?

RESPONDENT: It’s a good thing because if I continue to take it I will be healthy when I stopped taking treatment I started to get sick

INTERVIEWER: Besides taking treatment do you think there is another way of treating HIV according to you?

RESPONDENT: Can you please repeat the question.

INTERVIEWER: According to you, is taking this treatment that we are giving at the clinic and hospitals is it the only way or the is another way according to you?

RESPONDENT: Taking this treatment is the only way.

INTERVIEWER: Okay, is taking treatment important to you?

RESPONDENT: Yes.

INTERVIEWER: Why?

RESPONDENT: Because you won't be sick from this sickness but you will always be healthy.

INTERVIEWER: Okay, what will happen if you decide not to take treatment every day?

RESPONDENT: I will get sick and then my CD4 count will go down and then the will be problems from there.

INTERVIEWER: Okay, besides the clinic, have you take treatment from another clinic besides this one?

RESPONDENT: I use to take it back home in Limpopo.

INTERVIEWER: Back in Limpopo, is it a clinic or hospital?

RESPONDENT: A clinic.

INTERVIEWER: What is the name of the clinic?

RESPONDENT: [clinic name removed].

INTERVIEWER: Okay, let's look at [clinic name removed], how was your experience there?

RESPONDENT: It's in the rural areas, so it's not crowded and it doesn’t take time. It's not the same as here.

INTERVIEWER: So, your experience from there is different from here?

RESPONDENT: Yes.

INTERVIEWER: Is it good or bad?

RESPONDENT: It’s the same, they treat you the same.

INTERVIEWER: At that clinic, who assisted you at [clinic name removed] clinic, was it a nurse, admin clerk or someone else? Who was usually helping you when you were going there?

RESPONDENT: It’s the nurses.

INTERVIEWER: Okay, besides taking treatment for HIV, have you ever taken any treatment for HIV?

RESPONDENT: No.

INTERVIEWER: Nothing at all? Or maybe someone says to you “drink this it will help with HIV” or go to church to get prayed for?

RESPONDENT: No the is nothing.

INTERVIEWER: Okay, let's say you know someone who is HIV positive, would you recommend that person take treatment? Would you tell that person to take treatment?

RESPONDENT: Yes, I would encourage that person to take treatment.

INTERVIEWER: Why would you say to that person take treatment?

RESPONDENT: Because if you don’t take this treatment you will get sick and when you stop taking treatment it will be a risk.

INTERVIEWER: Okay, what else would you recommend to that person for them to stay healthy?

RESPONDENT: They must eat healthy food, exercise, and use condoms and continue to protect themselves.

INTERVIEWER: Okay, so to exercise and eating healthily will help as well?

RESPONDENT: Yes.

INTERVIEWER: Okay, since you are back on treatment are there any challenges that might make you stop taking treatment?

RESPONDENT: No.

INTERVIEWER: So you don’t have any challenges in terms of taking treatment now?

RESPONDENT: No.

INTERVIEWER: Okay, let’s look at opinions, you know people talk when it comes to HIV and you stay here in Alex, what are people saying when it comes to HIV?

RESPONDENT: People do talk, so the only thing you can do is just continue to take your treatment.

INTERVIEWER: So, that won't affect you?

RESPONDENT: No.

INTERVIEWER: Okay, in terms of side effects, because you are taking treatment and you have side effects, won't that affect you?

RESPONDENT: No.

INTERVIEWER: So, you take treatment at the same time, that won't affect you?

RESPONDENT: No.

INTERVIEWER: What can we do to help you to stay on treatment, what would you like us to do?

RESPONDENT: It's just that I saw that if I stop taking treatment I will end up being sick and I might end up with TB and end up dying.

INTERVIEWER: Okay, what would you like us to do as clinic staff for you to stay on treatment?

RESPONDENT: I don’t know maybe motivation.

INTERVIEWER: Motivation how? Calls? SMS?

RESPONDENT: Nurses, do come and visit you at your home of you didn’t come for your visit so that is fine.

INTERVIEWER: Okay, you said you came here yesterday?

RESPONDENT: Yes, my date was yesterday.

INTERVIEWER: How did you feel about the clinic visit, how was it?

RESPONDENT: It was alright because I got here before 9 in the morning and I left around noon.

INTERVIEWER: And the staff here, how was the treatment from them?

RESPONDENT: It was alright.

INTERVIEWER: Okay, looking at yesterday's visit and other previous visits, would you say it was good, bad or worse? Or it's still the same

RESPONDENT: Yesterday I came here early, I usually come here late.

INTERVIEWER: So, yesterday was a better visit?

RESPONDENT: Yes.

INTERVIEWER: Okay, what do you like about this clinic?

RESPONDENT: Its closer to where I stay.

INTERVIEWER: Is that the only thing?

RESPONDENT: Yes.

INTERVIEWER: Okay, what is it that you don't like about this clinic?

RESPONDENT: I don't know because I just come and take treatment and leave so, I don't know.

INTERVIEWER: If you can change your experience about re-starting treatment, what would that be?

RESPONDENT: Please repeat the question.

INTERVIEWER: If you could change your experience about re-starting treatment again, what would that be?

RESPONDENT: Like how they were treating me?

INTERVIEWER: Yes, like you use to take treatment before and then you stopped and now you are coming back treatment and on that day you come back to start treatment you experience something, what would that be? What could you change, maybe staff attitude or coming in late? Or you were happy about your experience of re-starting treatment

RESPONDENT: I experienced that coming early is the same because I leave late but when I come late I don't spend too much time at the clinic.

INTERVIEWER: Is that the only thing?

RESPONDENT: Yes.

INTERVIEWER: Okay, have you ever heard of a campaign called welcome back?

RESPONDENT: No.

INTERVIEWER: Or have you heard any adverts on radio or TV about coming back to care or from the clinic?

RESPONDENT: No.

INTERVIEWER: Okay, have you ever received a call from the clinic inviting you to come back to care?

RESPONDENT: No.

INTERVIEWER: So, they have never phoned this clinic? So, for you to come yesterday

RESPONDENT: Remember I took a transfer and I got a call from the clinic back home because they saw my date has passed and I didn't go.

INTERVIEWER: So, this clinic no one has ever called you?

RESPONDENT: No.

INTERVIEWER: Okay, did anyone visit you at your home to encourage you to come back to the clinic?

RESPONDENT: No.

INTERVIEWER: Okay, have you heard about the welcome back campaign, this is the last question?

RESPONDENT: No.

INTERVIEWER: You haven't heard?

RESPONDENT: Yes.

INTERVIEWER: Okay, do you have any questions or concerns?

RESPONDENT: No.

INTERVIEWER: Okay, in that case, we are done thank you.

RESPONDENT: Thank you.

Interview Type: IDI

SITE: Clinic A

INTERVIEWER: FB

TRANSCRIBER: FB

TRANSLATOR: FB

DATE OF INTERVIEW: 30/03/2020

DATE OF TRANSCRIPTION: 03/04/2020

TIME: 28:35

INTERVIEWER: Thank you once again. Like I said earlier the reason for this interview is to understand the use of treatment and the service you get from this clinic and also to understand how the welcome-back campaign is being implemented here and whether it is making a difference to clients who are returning to care like you. When we look at it, is it helping you and the community out there? Just a few things, if it happens while we are talking that you mention your name or anyone's name for that matter when I write this down I won't include it. What I will do I will use another name for instance if your name is Thembisile (not participant's real name) I will look for another name to use just to protect your identity. Whatever you tell me the staff working here won't know what you said, we will only come back to them and tell them what we found from patients. Firstly, just before I start with the questions for the interview we just want to understand who we are talking to. to understand you we just need to know your age, can you please tell me your age?

RESPONDENT: I am turning 35 this year.

INTERVIEWER: Okay, have you turned 35 already?

RESPONDENT: No, next month.

INTERVIEWER: Okay, your gender male or female so that we can understand?

RESPONDENT: Female.

INTERVIEWER: What is your marital status. Married, single?

RESPONDENT: Recently married.

INTERVIEWER: Recently married, congratulations. Okay, what is your highest level of education?

RESPONDENT: Its grade 11.

INTERVIEWER: Okay, what is your employment status?

RESPONDENT: I have been a receptionist…

INTERVIEWER: I mean currently.

RESPONDENT: Oh currently I'm unemployed.

INTERVIEWER: Okay thank you for that. So, the first question we want to understand is what service did you come here today at the clinic?

RESPONDENT: I came here to the clinic because it been some months since I defaulted and so, I got a call to come to the clinic and when I came they took my blood and they went through my file which is what they did first and then they told me that I can come today for counselling and this interview.

INTERVIEWER: Oh okay so, for today you only came for this only?

RESPONDENT: Yes, because I don't know what he meant when he said I should come back to him when I am done.

INTERVIEWER: Okay, out of all the different facilities that are here, why did you choose this one?

RESPONDENT: I didn't specifically choose this one, it's just that here at Alexandra there are different clinics and some are a bit far like there is one called Matakana, I came to this one because it is the nearest.

INTERVIEWER: Being the nearest is it the only reason or there are other reasons?

RESPONDENT: No, that is the only reason. I can't go to Marlboro whereas the is a clinic closer to me.

INTERVIEWER: Okay, thank you for that. Just to understand again have you ever stopped using treatment before?

RESPONDENT: This was my first time.

INTERVIEWER: Okay, so you are coming back to re-initiate on treatment again?

RESPONDENT: Yes.

INTERVIEWER: Okay, I was asking that so that I can understand the flow of questions. So, since you said you have stopped using the treatment for how long were you off treatment?

RESPONDENT: I think its six or seven months.

INTERVIEWER: You say its seven months. In these six or seven months, you were not taking treatment what were you taking?

RESPONDENT: Nothing.

INTERVIEWER: You weren't taking anything?

RESPONDENT: Nothing.

INTERVIEWER: You were not taking anything?

RESPONDENT: Yes.

INTERVIEWER: So, you were not attending any spiritual… Do you know some people will tell you that they believe in prayer?

RESPONDENT: No but I do go to church but for a different reason.

INTERVIEWER: Okay, like you said it's been six or seven months you have been off treatment, why did you stop?

RESPONDENT: Apparently I had to go the computer and there were some complications and so when I got there I think my form was not there and then there were some misunderstanding they told me that maybe I didn't bring it or whatsoever and then I just got pissed off and then I decided that it's better if I stopped.

INTERVIEWER: So, when you say a computer, what kind of computer is that?

RESPONDENT: here we do tests, so when we go the computer it is to reduce the queues here, so you go to the computer with your file and then they put everything there and then they give you your pills. You just come here to the clinic to do blood tests. When you collect your medication you don't come here.

INTERVIEWER: So, you stopped because of that misunderstanding?

RESPONDENT: Yes, of that misunderstanding.

INTERVIEWER: The person you had a misunderstanding with, was it with a staff member or?

RESPONDENT: I think it was a staff member because I was going to the computer as usual but unfortunately we didn't know that it expires and that you have to come here and renew it and then when you come here they tell you that you didn't fill it correctly, come back tomorrow and when you come back tomorrow there a lot of people, you know, that is why.

INTERVIEWER: Okay thank you for that I just wanted to understand whether it was with another client or what, thank you for that. Okay, before you stopped did you struggle with taking treatment?

RESPONDENT: No, I didn't.

INTERVIEWER: So, you never missed any doses?

RESPONDENT: Not at all.

INTERVIEWER: So, it was for that reason?

RESPONDENT: Yes, for that reason.

INTERVIEWER: Okay, is it your first time stopping treatment?

RESPONDENT: It is the first time.

INTERVIEWER: Okay, what made you say "you know what I have stopped treatment for six or seven months, I am going back to treatment"?

RESPONDENT: I think now it's because of my weight fluctuating and it's not the same anymore so I needed to know whether my CD4 count is it still fine and also my viral load is it still fine that is why. Because the more you stay away from treatment the more you get sick.

INTERVIEWER: Okay, making that decision that you are going back to treatment was it a difficult decision of you just woke up one and you decided that you are going back to treatment?

RESPONDENT: You know, it was difficult because at first, I brought my sister's child here and then I asked the case manager and he said that he was trying to call me because they wanted to keep me on the system and then he asked me whether I am ready I said yes I am and then he said I must come on Thursday and then I came on Thursday and then after that he told me I should come back today of this interview.

INTERVIEWER: Alright, thank you for that. As you are coming back to treatment did you discuss it with anyone, maybe your partner or family member?

RESPONDENT: I discussed it with my partner and my twin sister.

INTERVIEWER: Why did you discuss it with them specifically?

RESPONDENT: Because its people are usually talking to about this situation.

INTERVIEWER: So, both of them are aware of this situation?

RESPONDENT: Yes.

INTERVIEWER: Okay thank you for that once again, so the next questions are about your experience towards treatment, what was your experience when you tested for HIV and the first time you found out that you are positive?

RESPONDENT: The first time I was tested HIV I think I was 7 months pregnant. I think it was difficult because by that time the father of my child knew that he was positive but he didn't tell me, then I had to tell my mother, my father, basically my family apart from my husband because we haven't met by then. Then, I had to take treatment during pregnancy and I had a lot of ups and downs asking myself the question, why me? Why me? and the was nothing I could do I have to come back and take treatment and that was in 2014.

INTERVIEWER: Okay, do you feel that when you tested and found that you are positive, staff attitudes towards you how was it?

RESPONDENT: Staff attitudes, I wasn't here but I was at [name removed] Hospital.

INTERVIEWER: Staff attitudes there, how was it?

RESPONDENT: It was okay because I didn't have my children the natural way I had to had a C-section, I was told that they have to take blood, they took it and they gave me the medication for when you are pregnant, and then they also gave me the pill that you get when you get a child,

after that, I came here and then they told me here that it's still early for me to start with treatment but if I want to start taking treatment it's still fine they I can come and they will give me the treatment, they will continue to check my CD4 count and viral load and then I did that and that is when I started taking treatment.

INTERVIEWER: So, the staff attitude was oaky?

RESPONDENT: It was fine.

INTERVIEWER: I am trying to make this question as easy as possible, we all have opinions about certain things, what is your opinion about HIV treatment? Is it the best way to treat HIV or do you think there is another way to treat HIV?

RESPONDENT: I think it's still the best to treat it because you get counselling and when you default they call you back before they take you out of the system and they ask you what is the problem that is making you not take treatment, maybe it's your partner doesn't want you to take treatment or your family is neglecting you, you get counselling along those lines, so I think it's okay. Because they even take you to the machine where they can check high blood, sugar diabetes and they check everything.

INTERVIEWER: So, I am trying to make sense of the point of being neglected by your family, has it ever happen to you?

RESPONDENT: No because there was someone in the family who was HIV positive.

INTERVIEWER: So, they didn't have an issue?

RESPONDENT: No they didn't. The person who was positive I was very close with that person but didn't reveal that they are positive until they started getting sick that is when they revealed that they are positive.

INTERVIEWER: Okay, what do you understand about the importance of staying on treatment?

RESPONDENT: Staying treatment is important because you become healthy and you get to live longer and most black people don't believe that when you take the treatment you get to live longer; I don't know where do we get that.

INTERVIEWER: We were talking about the importance of taking treatment, why is it important to you?

RESPONDENT: It is very important because you can even eat healthily, take medication correctly, you can go to the gym even if you don't go to the gym there are certain things that you need to cut like alcohol and smoking.

INTERVIEWER: Alright, have you taken treatment from somewhere else apart from this clinic?

RESPONDENT: No.

INTERVIEWER: Is this the only clinic?

RESPONDENT: Yes, this is the only clinic.

INTERVIEWER: Earlier you mentioned that our black people don't believe in taking treatment for HIV, have you ever taken any other treatment for HIV before taking ARVs?

RESPONDENT: No.

INTERVIEWER: Okay, if you know someone, a friend or family members comes to you and tells you that they are HIV positive, would you tell or recommend that person to take treatment?

RESPONDENT: Yes, I do that. 90 per cent of my family know that I am positive and I am open about it if someone comes to and tells me that they are HIV positive, like my cousin she came to and told that she is HIV positive, I kept it to myself, she came to and told me that she positive and I told her that she should go to the clinic and get treatment but firstly you will get counselling and then you will get the treatment.

INTERVIEWER: Okay, so you did recommend?

RESPONDENT: Yes, I did.

INTERVIEWER: Okay, besides treatment what else would you recommend to that person?

RESPONDENT: I don't think I would recommend anything because you can't say to someone go to this specific person and douche. You will be killing that person. Rather they come to the clinic.

INTERVIEWER: Okay, just to understand again it's the first time that you are coming back to treatment?

RESPONDENT: Yes.

INTERVIEWER: To help someone to stay on treatment, what can we do? Looking at the whole clinic…

RESPONDENT: To keep them on treatment?

INTERVIEWER: Yes, looking at you, for instance, what can we do to help you to stay on treatment? Let's look at staff attitudes, what can we do about that?

RESPONDENT: Like the case manager he is doing a great job like he knows how to talk to people who have defaulted from treatment and those who are coming back to the clinic. He sits down with you and tries to understand what is going on, why did you leave the treatment and why are you coming back again, how are you feeling now? I think it is the best way to do it.

INTERVIEWER: Alright, in terms of the health system that we have, what can we do?

RESPONDENT: health system we have, I think they should tell people directly that if you do this and that it will harm your body this way and that way.

INTERVIEWER: Okay, thank you for that. We are left with few questions and then we are done. Now we are going to look at today's visit. How do you feel about today's visit? Before you

answer that one I just want to understand how far are you with your visit today, have you seen anyone apart from the case manager today?

RESPONDENT: Yes.

INTERVIEWER: Who’s that person?

RESPONDENT: A nurse.

INTERVIEWER: How was the nurse?

RESPONDENT: She was okay because before I went to the case manager she spoke to me for like an hour trying to understand what happened and she was worried that I have been taking my medication and I stopped and she wanted to understand so that she can talk to me like the case manager did.

INTERVIEWER: Okay, what about waiting time?

RESPONDENT: I waited for some time today but normally I don’t.

INTERVIEWER: Okay, comparing today’s visit with other visits, how was it? Let's rate it out of 10?

RESPONDENT: I would give it an 8?

INTERVIEWER: Why an 8?

RESPONDENT: That waiting period only.

INTERVIEWER: Okay, its fine, what did you like about this clinic?

RESPONDENT: I like the fact there is a doctor to patient confidentiality.

INTERVIEWER: Okay, as much as there is something that you like, there is also something that you don’t like, what is that?

RESPONDENT: What could it be?

INTERVIEWER: It can be anything, it can be the waiting time, how they greeted you at the doctor or it can be anything

RESPONDENT: Some people are always grumpy and we don’t know what happened but then some people come and sit down with you and ask you questions and maybe I don’t answer the way you expected and then you snap that is what I don’t like.

INTERVIEWER: Has that ever happened to you here?

RESPONDENT: No.

INTERVIEWER: Okay, what can you change about your experience on re-starting treatment today?

RESPONDENT: That when you work with people it doesn't mean that I got here at 8 in the morning I should leave here at 2 in the afternoon, we understand that they should take their tea and all that but they shouldn't take their time, maybe I as well let's say I am working maybe I told my employer that I will be at work by 10 and then I get here at 7 in the morning and I leave at noon, it's not fair. Then I should take a doctor's note and when I do that people will say I didn't want to go work.

INTERVIEWER: Thank you for that. Okay, now this is the last section, depending on how you answer them. We are almost done. So, these questions are about Welcome Back Campaign like I said earlier, about Welcome Back Campaign have you heard anything about it on the radio or seen adverts on it about coming back to care or heard about it here at the clinic?

RESPONDENT: No, I just came by myself.

INTERVIEWER: Okay about the welcome back campaign?

RESPONDENT: No I have never but the case manager did give me a hint.

INTERVIEWER: Okay, so you heard about it from someone here in the clinic?

RESPONDENT: Yes.

INTERVIEWER: Have they ever called here at the clinic encouraging you to come back to care?

RESPONDENT: Yes.

INTERVIEWER: About that call, how did you feel? Did you feel that they are invading your privacy or how did you feel?

RESPONDENT: I felt good because sometimes when you get that call from the clinic, the first thing that comes to mind is "what were you doing when you stopped taking treatment" those kinds of things but I didn't get that.

INTERVIEWER: Okay, did anyone visit you at home…?

RESPONDENT: No.

INTERVIEWER: Okay, just to encourage you to come back to care?

RESPONDENT: No.

INTERVIEWER: Okay, just to repeat, have you ever heard about the welcome back campaign?

RESPONDENT: No I have never. Apart from the case manager.

INTERVIEWER: Okay, When the case manager called you what did he say?

RESPONDENT: He called me and then he explained what he was doing and after talking, he said to me I should come Tuesday because there are people who want to talk to me.

INTERVIEWER: Okay, that call from the case manager did it influence you to come back to treatment?

RESPONDENT: Not really, because I was thinking of doing that.

INTERVIEWER: So, it played a bit of encouragement?

RESPONDENT: Yes, a bit.

INTERVIEWER: Okay, do you have any question or comment or you would like some clarity?

RESPONDENT: So, this campaign will help in terms of visiting people who are afraid of coming to the clinic because there are people who are afraid of coming to the clinic? Will it be a campaign of doing a door to door?

INTERVIEWER: Okay, I will make an example of where I come from in Soweto because I know they heard it this past Saturday, so what they did in Soweto they went to a certain place and put tents there and they had a session about welcome back campaign if they have to do a door to door then they will have to do it. This campaign's about encouraging people, you tested before and the results came back positive and you started treatment, this is what we are promising you when you come back to treatment.

RESPONDENT: Okay.

INTERVIEWER: If there are no further questions we are done, thank you

RESPONDENT: Thanks.

Interview Type: IDI

SITE: Clinic C

INTERVIEWER: FB

TRANSCRIBER: FB

TRANSLATOR: FB

DATE OF INTERVIEW: 16/03/2020

DATE OF TRANSCRIPTION: 19/03/2020

TIME: 15:38

INTERVIEWER: Okay thank you once again, Lime I said earlier we are doing this interview we are trying to see the campaign that is being done called Welcome Back Campaign, so Welcome Back Campaign it's a campaign to welcome back people who were taking treatment before and they stopped, now they are coming back. What we are trying to see is if this campaign is being implemented correctly, is it making a difference to you, the community and the staff working here. We also want to improve the service as well. Most of the questions they are about you and if you feel uncomfortable answering certain questions you will let me know and we will skip that question. If you include your name or the staff member's name by mistake, when I write this down I won include those names or I will use a different name. everything you tell me the clinic staff won't know what you said. The only thing they will know is that when we come back and give them the results we will tell them that we have interviewed these numbers of patients and this is what they are saying. So, the first few questions we just want to understand about you, can you please tell me your age, how old are you?

RESPONDENT: 33.

INTERVIEWER: Okay, are you married or not?

RESPONDENT: I am not married.

INTERVIEWER: Do you have a partner?

RESPONDENT: Here I don't have a partner.

INTERVIEWER: Okay your partner is back home?

RESPONDENT: No, we broke up with that lady.

INTERVIEWER: Okay, what is your highest level of education?

RESPONDENT: Its Grade 11.

INTERVIEWER: What is your employment status?

RESPONDENT: I am doing temporary work.

INTERVIEWER: Okay, it's fine to thank you for that. Can you please tell me what service did you come here for at this clinic?

RESPONDENT: I came to collect treatment for HIV.

INTERVIEWER: You stay here at Alex right? There are many clinics here, why did you choose this one?

RESPONDENT: When I checked I saw that the clinic on 18th avenue they are renovating it, so I came here in 2007 to test and that is when I found out that I am positive and so, I decided that I should follow the rules and see how it goes. When I went back to 18th avenue they told me that people who stay at Stwetla need to go to clinic C and then I got a job and in that job, I wasn't getting paid well and then met a couple of people at work and we started to do wrong things and then they fired us at work.

INTERVIEWER: Okay, so why did you choose this clinic?

RESPONDENT: This clinic is closer to me. I don't want to go far and pay money that is why I chose this clinic.

INTERVIEWER: That is why you chose this clinic because it's closer to you?

RESPONDENT: Yes.

INTERVIEWER: Okay, can you please tell me when did you find out that you are HIV positive?

RESPONDENT: In 2007

INTERVIEWER: And when did you start to take treatment?

RESPONDENT: In 2007.

INTERVIEWER: Okay, and when did you stop taking treatment?

RESPONDENT: I stopped last year in September.

INTERVIEWER: Okay and when did you start it again?

RESPONDENT: This month.

INTERVIEWER: Okay, that time you had stopped between September last year and February this year, is the anything you were taking at that time?

RESPONDENT: Yes, I got someone whole I was in prison who was also HIV positive and I saw him taking this treatment and I went to him and told him that I have a problem and this guy asked me what was the problem, I told him that I am positive and home is far and I have to take blood and I can't get home at the moment, my sister use to go and collect treatment for me while I was at work so now I can't get my treatment and this guy said to me that he has plenty of these

pills. The thing is I was afraid of telling the nurses there that I am positive. This guy gave me one container and told me I will see how I take them.

INTERVIEWER: Okay, so you were taking someone else's treatment?

RESPONDENT: Yes.

INTERVIEWER: Okay, that is the only thing you were taking?

RESPONDENT: Yes.

INTERVIEWER: Okay, since you said you were not able to come to the clinic and collect your treatment was because you were arrested?

RESPONDENT: Yes.

INTERVIEWER: Is that the only reason?

RESPONDENT: Yes.

INTERVIEWER: Okay, you said you found out in 2007 that you are positive and you started taking treatment that same year until last year September. During that time, you were taking treatment, you were taking it every day?

RESPONDENT: Yes. I never made a mistake, every day I was taking them.

INTERVIEWER: Okay so, you stop taking treatment last year September until this year in February, was it your first time stopping taking treatment or have you stopped before?

RESPONDENT: Yes, when I left prison and came back to Joburg I remembered that I have this problem and I wasn't sure if I should come back and whether they would swear at me or what, they didn't swear at me they told me that I should come and see a counsellor and even when I got the piece job at Randburg like today if like I came today I told the counsellor that I would be here at 11 in the morning and then 12 my lunch is over, so I came during this time.

INTERVIEWER: Okay, thank you for that. So, you came back because you know that you need to take treatment and that is why you have started taking treatment again?

RESPONDENT: Yes, the counsellor told me that if I have to leave and go to work somewhere I should tell them and they can give me treatment for 3 months and then if I have to take blood then I have to come to Joburg. But If I am not taking blood then they would send me my 3-month medication and then if I have to take blood I would have to come back to Joburg to come to the clinic.

INTERVIEWER: Okay, when you came back last month to restart treatment did you discuss it with anyone that you are coming back to take treatment or you just decided that you are coming back to treatment without discussing it with anyone?

RESPONDENT: I came back here and I remembered that I need to come to the clinic because now I am out of prison.

INTERVIEWER: Okay, have you ever taken treatment from another clinic?

RESPONDENT: Yes, it's here and 18th avenue clinic.

INTERVIEWER: Oh and then on 18th avenue they sent you here?

RESPONDENT: On 18th they said I should come to this clinic because it's closer to me. We started there because they said this clinic is being renovated and then they said because the clinic is complete we can come back here.

INTERVIEWER: Okay, if someone comes to you and tells you that he or she is positive, would you recommend that person to take treatment?

RESPONDENT: To come and collect treatment for me?

INTERVIEWER: No, that person comes to you and tells you that they are positive, would you encourage that person to go and take treatment?

RESPONDENT: Yes, I would.

INTERVIEWER: Okay, why?

RESPONDENT: You now they say we shouldn't be afraid of telling people that you are positive but we should guide people and encourage them in taking treatment because it's their life, so I will tell that person that.

INTERVIEWER: Okay, since you are back now to take treatment, do you think there is anything that could stop you from taking treatment?

RESPONDENT: No.

INTERVIEWER: Okay, that time you tested…let's go back to 2007 a bit, that time you test and you found out that you are positive how did you feel?

RESPONDENT: The first time when I tested, I was sitting at home and then I thought let me go and test because there are these illnesses go around, so I went to test Diabetes and HIV. They said my Diabetes is fine and then I tested HIV and they told me that I am positive. My heart was broken but I told myself that this is not a problem let me deal with it now because if I don't then I will die. I thought about not taking treatment but managed to convince myself otherwise and then I told myself that it has happened I can't change the past let me take the treatment.

INTERVIEWER: Thank you for that. What comes to your mind when you think about treatment?

RESPONDENT: I think about my life. If I take it for 5 years maybe it would give me 5 more years again.

INTERVIEWER: Okay so, treatment is a good thing?

RESPONDENT: Yes.

INTERVIEWER: What do you like about this clinic?

RESPONDENT: They treat me well.

INTERVIEWER: That time you came to collect your treatment, how was your visit? Was it good, better or worse?

RESPONDENT: Me?

INTERVIEWER: Yes.

RESPONDENT: I don't have a bad record of this place ever since I started coming here.

INTERVIEWER: Okay, have you ever heard about a campaign on the radio during adverts even on the radio?

RESPONDENT: Yes.

INTERVIEWER: Okay, have they ever called you here at the clinic?

RESPONDENT: No.

INTERVIEWER: So they have never called you?

RESPONDENT: They only called me today to come and see a counsellor.

INTERVIEWER: When you heard or seen the advert about welcome back, did it encourage you to come back to treatment?

RESPONDENT: Yes, it did. You know when you see someone who has been taking treatment and they stop you will see how they look, so I am afraid of stopping treatment because I would fall back and become sick and when you come back at the clinic they will tell you that you must take treatment until you die.

INTERVIEWER: Okay, can you please tell me, when did you come to collect your treatment?

RESPONDENT: It was last week Wednesday.

INTERVIEWER: Okay, that time you came here on Wednesday how was it? Did you wait a long time or on?

RESPONDENT: I didn't wait that long.

INTERVIEWER: You didn't wait that long, so you were happy with the visit?

RESPONDENT: Yes.

INTERVIEWER: Okay, do you have any questions or something you would like us to discuss?

RESPONDENT: I just have one thing. I came to you today to have this interview with you and this interview is good because it encourages me to stay on treatment. When I think about not taking treatment I would be killing myself. I hear you loud and clear when we are talking.

INTERVIEWER: Yes, we just want you to stay on treatment.

RESPONDENT: Yes. Thank you very much.

INTERVIEWER: Thank you, if you don’t have any question or concern we are done.

RESPONDENT: Thanks.

Interview Type: IDI

SITE: Clinic G

INTERVIEWER: FB

TRANSCRIBER: FB

TRANSLATOR: FB

DATE OF INTERVIEW: 11/06/2020

DATE OF TRANSCRIPTION: 15/06/2020

TIME: 18:27

INTERVIEWER: This is a campaign that is run by the government called Welcome back campaign, so this campaign wants to welcome back patients who were taking treatment before at the clinics, so we want to see how is the service being improved, and how do they feel about returning to care. So, the questions I am going to ask are about that and you as well. The first questions I am going to ask are about you, can you please tell me how old are you?

RESPONDENT: 38 years old.

INTERVIEWER: Are you married or not?

RESPONDENT: No I am not married.

INTERVIEWER: What is your highest level of education?

RESPONDENT: Grade 12.

INTERVIEWER: Okay, at the moment are you employed or unemployed, or are you self-employed?

RESPONDENT: I am self-employed.

INTERVIEWER: Okay. No problem. Can you please tell me when was the last time you went to the clinic?

RESPONDENT: Before lockdown started. I don't remember the exact date.

INTERVIEWER: Okay, so the last time you went to the clinic was around March?

RESPONDENT: Yes.

INTERVIEWER: Okay, when you went there in March, what was the reason for the visit?

RESPONDENT: I was collecting my treatment.

INTERVIEWER: Alright, as you went to Clinic G, why did you choose Clinic G?

RESPONDENT: Can you please repeat the question.

INTERVIEWER: As you went to Clinic G, why did you choose it?

RESPONDENT: It's because I stay closer to it.

INTERVIEWER: Okay, it's because you stay closer to it. is that the only reason?

RESPONDENT: Yes, because I can't go far because if I don that it means I will struggle to go there if I don't have transport.

INTERVIEWER: Alright. So when you go to Clinic G you just walk?

RESPONDENT: Yes, it's a walking distance.

INTERVIEWER: Okay, can you please tell me sir, has it ever happened that you stopped taking treatment?

RESPONDENT: Yes, it has happened before.

INTERVIEWER: Okay, when was that?

RESPONDENT: Before I went to Clinic G I was taking my treatment in Pretoria and then I moved this side. Another thing is that the person I took the transfer letter from wrote that I am a troublesome person and all those kinds of things. So when I went to Clinic G and they saw those things they said they won't give me a full month's treatment but I have to come every week to come and take my treatment and they need to monitor me and then once they have done that I stopped and I didn't take treatment for the whole year and I only went back when I was sick and I was not feeling well and then now I am doing everything in a right way.

INTERVIEWER: So you went back because you were sick?

RESPONDENT: Yes.

INTERVIEWER: Okay, that time you were not taking treatment for the whole, is the anything you were taking?

RESPONDENT: At that time, I wasn't taking anything but sometimes I would take vitamins.

INTERVIEWER: Okay, so you were taking vitamins only?

RESPONDENT: Yes, I was taking vitamins only.

INTERVIEWER: And then the reason for you to stop taking treatment was that because they were not giving you a full dose of treatment and then you had to go back and get them that is why you stopped?

RESPONDENT: The thing is that was only for one day, when I got there with the transfer letter and I produced it, it was not that I wasn't welcomed but it was because of the things that are written in the transfer because what had happened is that when I was still staying in Pretoria you would find that when it was my appointment date and I couldn't come on that day and I could get there on time, I would just get there when its time just to collect, so I saw that because I am this side, its better I take the transfer from that side and come to this side, so, when I took the

transfer they had already written in it and then when I got there and produced my transfer the lady I gave it to called everyone and said that " here he is they say he is troublesome", and they were talking things I didn't understand.

INTERVIEWER: Okay because of what was written in the transfer letter contributed to making you stop taking treatment?

RESPONDENT: Yes, I can just say that I couldn't just go and take them every four to five days and I was used to taking them just once for the whole month. Because you find that every 3 days I have to go and collect and with work they won't just allow you every time to go and taking day offs every week.

INTERVIEWER: Okay, and then that time when you said that you are going back treatment, did you discuss it with anyone or you just decided all by yourself?

RESPONDENT: I didn't discuss it with anyone. What had happened is that I was circulating in all these clinics, because I remember I once went to Paulshof and when I got there I told them that I am this side and then they gave me once and when I went back they said they want a transfer letter but because of what I was written I couldn't produce it and then I also went to Parkhurst I also collected there but because I couldn't collect in these places because they knew that I collect without a transfer letter and in all these places they wanted a transfer letter and then I ended going back to Pretoria and explained everything to them because I feel that they are the ones who affected me with what they wrote in the transfer letter. Because when I get they don't make me feel comfortable because they call each other and they don't make me feel welcomed.

INTERVIEWER: Okay thank you for that. Can you please tell me when did you find out that you are positive the first time?

RESPONDENT: The first time it was… now we are in 2020 so I think it was in 2017.

INTERVIEWER: Alright, when you found out that you are positive in 2017 how did you feel?

RESPONDENT: When I found out I was in the hospital because I was sick, I didn't know how I feel but it felt like I am losing my mind a little bit.

INTERVIEWER: Okay so, when they tested you and you found out that you are positive, how did you feel?

RESPONDENT: It didn't it, I accepted like even now I don't have a problem, I accepted that this thing is there.

INTERVIEWER: Okay, since you are back on treatment, is there anything that is going to disturb you from taking treatment?

RESPONDENT: I don't think so. Because even now they gave me treatment in March, they gave me something that will last me until July. I will go back in July. As long I have it on my side I am fine.

INTERVIEWER: Okay and then what can we do to support you to stay on treatment?

RESPONDENT: I don’t think I am struggling as that thing happened before. You know when you get into a place where you feel like you are not welcomed and that thing does discourage you as well and sometimes just need someone to sit down with you and talk to you and you can see that you are welcomed and here when I went the second time I appreciate their welcome, it’s not like the first time I went there I don’t know maybe I met a wrong person.

INTERVIEWER: And when it comes to treatment, what do you think about it, is it a good thing or a bad thing?

RESPONDENT: the thing is when I first started it wasn’t treating me well like my face was itching, I wasn’t the same anymore, there were changes, and the continue giving it to and then a doctor there at Clinic G told me that I should stop using Shower gels and all these things and then I stopped and it stopped like I appreciate the way he was advising me.

INTERVIEWER: Okay, if let's say a family member or friend comes to you and tells you about their status, would you tell them to take treatment?

RESPONDENT: Yes, I would because I experienced it that if you don’t take treatment yourself you are killing yourself, there would be a time where you would be strong and the would also be a time where you would feel like you can’t.

INTERVIEWER: Okay, and then the last time you went to the clinic before Lockdown, how was the service?

RESPONDENT: I can say 100%. I don’t know the first time when I got there maybe it was the person I first encountered there but it was a while back maybe it was in 2018.

INTERVIEWER: Yes, I mean the last time you went there before lockdown, how was the service?

RESPONDENT: It was alright. I appreciate everything, everything is perfect.

INTERVIEWER: Okay, what do you like about Clinic G?

RESPONDENT: Everything, like they are always reminding me about everything that is happening.

INTERVIEWER: Okay and then what is it that you don’t like about Clinic G?

RESPONDENT: Everything is fine.

INTERVIEWER: Everything is fine. Can you please tell me have you ever heard of a campaign called Welcome Back on the TV or radio?

RESPONDENT: No.

INTERVIEWER: Have you heard about it at the clinic?

RESPONDENT: No.

INTERVIEWER: At Clinic G have they ever called and encouraging you to come back to the clinic?

RESPONDENT: Yes, they always call me and update me about everything and they remind me about my next appointment.

INTERVIEWER: Okay, when they call you, like when they remind you about your next appointment, how do you feel about those calls?

RESPONDENT: They make me feel happy because you think only these things happen only in private clinics, another thing is that when it comes to treatment from government clinics it's bad but when it does something right, it's good.

INTERVIEWER: Okay we have come to the end of the interview thank you so much.

RESPONDENT: Thank you.
